# CSI acquisition in RIS-assisted mobile communication systems

**DOI:** 10.1093/nsr/nwad127

**Published:** 2023-05-05

**Authors:** Yu Han, Wankai Tang, Xiao Li, Michail Matthaiou, Shi Jin

**Affiliations:** National Mobile Communications Research Laboratory, Southeast University, Nanjing 210096, China; National Mobile Communications Research Laboratory, Southeast University, Nanjing 210096, China; National Mobile Communications Research Laboratory, Southeast University, Nanjing 210096, China; Centre for Wireless Innovation (CWI), Queen’s University Belfast, Belfast BT3 9DT, UK; National Mobile Communications Research Laboratory, Southeast University, Nanjing 210096, China

**Keywords:** channel estimation, hybrid RIS, implicit CSI, RIS

## Abstract

Reconfigurable intelligent surface- (RIS) assisted mobile communication is a promising technological paradigm thanks to the attractive advantages of low cost and flexible control of electromagnetic waves. However, the low-cost features of RISs entail some fundamental challenges to the acquisition of channel state information (CSI), which is essential for the optimal RIS design. To tackle this problem, there have been extensive studies on CSI acquisition in RIS-assisted mobile communication systems, from the perspective of architectural improvement as well as specific mathematical solutions. This article aims to overview the existing works on CSI acquisition in RIS-assisted mobile communication systems.

## Teaser

This paper gives an overview of the work on CSI acquisition in RIS-assisted mobile communication systems with a focus on various categories of RISs, implicit and explicit CSI acquisition, and different channel estimation targets and algorithms.

## INTRODUCTION

The great success of multiantenna techniques in the last three generations of mobile communications makes us more clearly recognize the importance of exploring resources in the spatial domain. Future mobile systems will be equipped with even more antennas to further exploit the spatial degrees of freedom. However, large-scale antenna arrays will create formidable challenges from a cost and power consumption perspective. During recent years, reconfigurable intelligent surfaces (RISs), also known as programmable metasurfaces, have experienced a rapid development [1–4]. A RIS is a programmable surface composed of massive units whose electromagnetic responses can be artificially controlled in a real-time manner. It can be regarded as a reduced version of a large antenna array without signal transmission or reception modules, but with the advantages of low cost and low power consumption. Therefore, for the purposes of exploiting the spatial degrees of freedom and contributing to green communications, RISs have been introduced in mobile systems to produce a customized electromagnetic propagation environment and then improve the communication service quality.

In the early study of RIS-assisted mobile communications, a RIS is generally deployed in areas where users suffer from severe signal degradation in order to provide a complementary link to reflect an incident signal towards a desirable direction and to sustain the communication service. Compared with traditional relays, a RIS can greatly reduce the hardware cost and power consumption and simultaneously enable full duplex operation. Apart from communication recovery, a RIS can also improve the wireless channel by increasing the channel rank, and even enable emerging applications, such as localization and sensing [5–8]. The precondition of harvesting the gain provided by RIS is the acquisition of channel state information (CSI), especially in the RIS link. There have been extensive studies on CSI acquisition in RIS-assisted mobile systems. This article makes a review of the state of the art.

**Figure 1.**
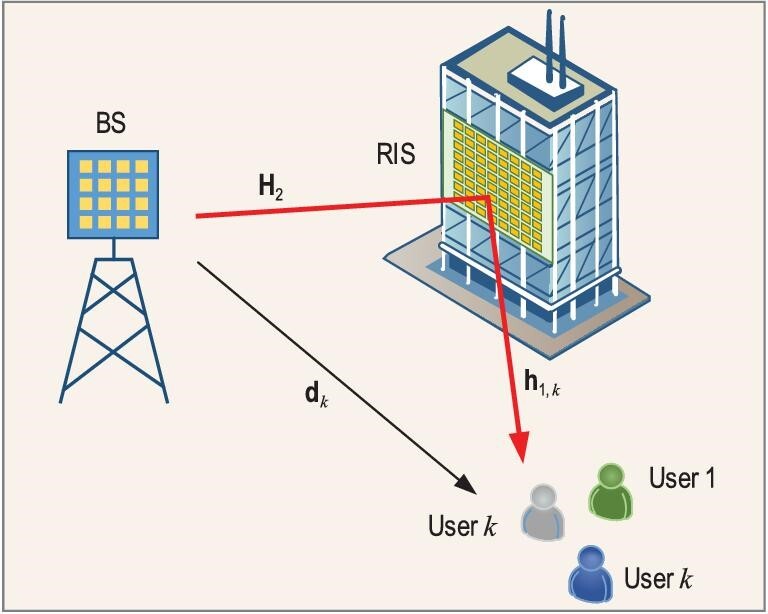
RIS-assisted mobile communications, where a RIS provides an additional link to improve the service quality.

Our review is based on the following system model, as shown in Fig. 1. The mobile system works in the time division duplexing (TDD) mode. The base station (BS) is equipped with *M* antennas, serving *K* single-antenna users in the cell. Suppose that user *k* is at the cell edge or in the shadow of a building, suffering from severe signal degradation. Denote the channel between the BS and user *k* as ${\bf d}_k \in \mathbb {C}^{M\times 1}$. Then, **d**_*k*_ is of poor quality and has weak power. In order to enhance the receiving signal strength at user *k*, a RIS is deployed between the BS and user *k* to provide an assistance link. The RIS is composed of *N* units, each with the ability to reflect the incident signal towards a desired direction by adjusting the phase of the signal. Denote the channel between the BS and RIS and the channel between the RIS and user *k* as ${\bf H}_2\in \mathbb {C}^{M\times N}$ and ${\bf h}_{1,k} \in \mathbb {C}^{N\times 1}$, respectively. In the data transmission stage, the signal received at user *k* can be expressed as


(1) \begin{eqnarray*} r_k = \sqrt{P} ( {\bf d}_k^T+{\bf h}_{1,k}^T{\boldsymbol \Phi }{\bf H}_2^T )\sum _{k=1}^K {\bf f}_k x_k + n_k, \end{eqnarray*}

where *P* is the transmission power at the BS, **Φ** = diag{**v**} is the phase shift matrix at the RIS, ${\bf v}=[e^{j\theta _1},\ldots ,e^{j\theta _N}]^T$ satisfies θ_*n*_ ∈ [0, 2π], ${\bf f}_k\in \mathbb {C}^{M\times 1}$ is the precoding vector to user *k, x_k_* is the signal sent to user *k* and *n_k_* is the additive complex Gaussian noise. In addition to the direct link, ${\bf d}_k^T$, which is of poor quality, the RIS provides a controllable supplementary link ${\bf h}_{1,k}^T{\boldsymbol \Phi }{\bf H}_2^H$. The power of ${\bf h}_{1,k}^T{\boldsymbol \Phi }{\bf H}_2^H$ can be significantly enhanced by properly adjusting **Φ**, which can be realized when CSI is available.

In the context of CSI acquisition, this article starts from an introduction of the current categories of RISs, including passive RISs, hybrid RISs and active RISs. Based on the type of CSI to be acquired, implicit and explicit CSI acquisition methods are summarized, respectively. For explicit CSI especially, the methods to separate the direct link and the RIS link channels are first reviewed, involving high-overhead linear cascaded channel estimation in the RIS link. The channel features that can help reduce the cascaded channel estimation overhead are then articulated. Afterwards, the methods to estimate individual channels in the RIS link for different categories of RISs are reviewed. Then, the widely used channel estimation algorithms for explicit CSI acquisition are summarized. Finally, we present two open problems that will arise with the occurrence of new architectures and application scenarios.

*Notation.*—Letters in normal, lowercase bold and uppercase bold fonts are used for scalars, vectors and matrices, respectively. The superscripts ( · )^*T*^, ( · )^*H*^ and ( · )^†^ return the transpose, conjugate transpose, and pseudo-inverse, respectively; | · | and ‖ · ‖ return the absolute value and the modulus, respectively; $\mathbb {E}\lbrace \cdot \rbrace$ denotes taking the expectation. The notation ⌈ · ⌉ represents rounding up the value; [ · ]_*m*, :_, [ · ]_:, *n*_ and [ · ]_*m, n*_ extract the *m*th row, the *n*th column and the (*m, n*)th entry of a matrix, respectively. Finally, ‘${}\circ $’ returns the column-wise Kronecker product.

## CATEGORIES OF RISs

During the last few years, a variety of RISs have appeared, each with its unique structure and functionalities. In this section, we introduce the existing categories of RISs and make a comparison among them. It should be clarified that the definitions of passive, hybrid and active RISs in this article are determined on the basis of existing RISs that have been published in the literature. With the emergence of new RIS architectures, the definitions and categories of RISs will vary.

### Passive RIS

We say that a RIS is passive when it is equipped with neither a power amplifier (PA), low noise amplifier nor signal transmission or reception radio-frequency (RF) chains. Here, passive does not mean that no input power is required by the RIS. A low-level external voltage is still needed to control the phase shift for signal reflection.

**Figure 2.**
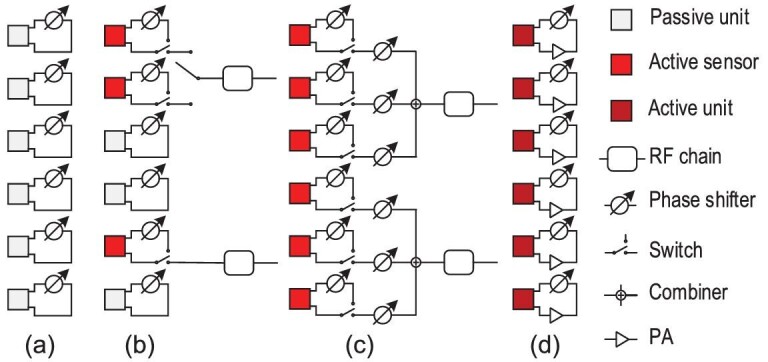
Different categories of RISs, including the (a) passive RIS, (b) selection-type hybrid RIS, (c) beamforming-type hybrid RIS and (d) active RIS.

The most widely used passive RIS is the reflective-only passive RIS shown in Fig. 2(a) [9–17]. It is usually located on the roof or facade of a building, reflecting signals in front of it. Thus, the reflective-only passive RIS is also named the intelligent reflecting surface (IRS) [11,12]. When an IRS is introduced to assist the mobile communication, the individual channels **H**_2_ and **h**_1, *k*_ are cascaded together through **Φ**. The effective channel at the IRS link is **H**_2_**Φh**_1, *k*_. Since signal processing modules are absent at the IRS, we can only estimate the channels at the BS or user side.

Apart from IRS, some passive RISs have the ability to refract the incident signal, including the refractive-only passive RISs and the simultaneous refractive and reflective passive RISs. A refractive-only passive RIS is also known as a reconfigurable refraction surface (RRS) [18,19]. It is generally located at the BS, acting as a phased antenna array. A RRS refracts the signal transmitted from the feed at the BS and controls the refraction direction of the signal by adjusting its phase. Different from the IRS-assisted system, where the IRS provides an additional link apart from the direct link, in a RRS-assisted system, only the BS-RRS-user link exists.

A passive RIS offering both refraction and reflection [20–22] is referred to as an intelligent omni-direction surface (IOS) or a simultaneously transmitting and reflecting RIS. In an ideal scenario, an IOS can replace window glass to enable signal reflection in front of the window and signal refraction across the window. Then, two users that are at different sides of the IOS can be served at the same time. Channel estimation in RRS and IOS-assisted systems is still in its infancy, and is thus beyond the scope of this review paper.

### Hybrid RIS

To address the challenge of channel estimation in passive RIS-assisted mobile systems caused by the absence of signal processing capabilities, a semi-passive RIS, also known as a hybrid active and passive RIS, has been proposed in [23–28]. In this paper, we use a hybrid RIS for the simplification of expression. Hybrid RISs are equipped with signal reception or even transmitting RF chains, which can be connected with active sensors.

Each active sensor has two modes, including the antenna mode and the reflection mode. In the antenna mode, active sensors can receive pilots sent from the BS and users or even transmit pilots to them. Thus, channel estimation is supported at the RIS side. Denote by ρ_*n*_ ∈ [0, 1] the power ratio of the received signal over the incident signal on the *n*th unit; ρ_*n*_ = 0 and 1 represent the scenarios of the incident signal being completely reflected and received, respectively. When 0 < ρ_*n*_ < 1, signal reflection and reception happen simultaneously. The practical value range of ρ_*n*_ is determined by the hardware structure of the hybrid RIS.

In a hybrid RIS, a few or even all passive RIS units can be replaced by active sensors. Assume that *N*_a_ out of *N* units are selected and replaced by active sensors, and that the number of signal reception RF chains is *N*_RF_. Generally, we have *N*_RF_ ≤ *N*_a_ ≤ *N*. Thus, a hybrid RIS can perform channel estimation and also maintains the RIS’s advantages of low hardware cost and low power consumption.

Based on the connection type between RF chains and active sensors, we further divide hybrid RISs into two sub-categories. One is the selection-type hybrid RIS, in which at one time instance, each RF chain is connected with only one active sensor, as shown in Fig. 2(b). If *N*_RF_ < *N*_a_ then a switch is further needed to make a selection between a RF chain and its possibly connected active sensor. The other is the beamforming-type hybrid RIS, in which each RF chain is connected with multiple or even all active sensors simultaneously. Analog beamforming is performed before signals received by the active sensors are combined at the RF chains, as illustrated in Fig. 2(c).

### Active RIS

Figure 2(d) illustrates an active RIS, whose units are all active. Note that an active unit is different from an active sensor. An active unit has only one mode, i.e. the reflection mode. No RF chain is connected with an active unit. It should be connected with a PA to enhance the incident signal power [29–32]. The motivation to introduce active RISs is to overcome the multiplicative pathloss in the BS-RIS and RIS-user channels. However, an active RIS still works solely in the reflection mode and it is not equipped with signal reception RF chains. It cannot transmit or receive signals, and thus does not support channel estimation at the RIS side. Channel estimation in an active RIS-assisted system is the same as that in an IRS-assisted system.

No matter which structure the RIS has, in the data transmission stage, the RIS is switched to the reflection and/or refraction mode and improves the mobile communication quality by adjusting the signal phase. RIS phase shifting operates like analog beamforming in millimeter wave hybrid beamforming systems. In order to optimize the phase shift, acquisition of implicit or explicit CSI at the RIS is indispensable.

## IMPLICIT CSI ACQUISITION

If the design of the RIS phase shift in the data transmission stage does not rely on the explicit CSI, i.e. the full channel **h**_1, *k*_ or **H**_2_ or their cascaded version, then we can simply acquire the implicit CSI. Implicit CSI reflects the channel condition. It can be a beam index, a discrete phase index, etc.

### Beam training

The original motivation to introduce RISs is to enhance the total channel power. Recalling Equation (1), the optimal $\boldsymbol {\Phi}$, or, equivalently, the optimal $\bf v$, should maximize $\Vert {\bf d}_k^T+{\bf h}_{1,k}^T{\boldsymbol{\Phi} }{\bf H}_2^T \Vert ^2$. Following the beam training approach in hybrid beamforming systems, a predefined RIS phase shift codebook can be utilized, and the optimal RIS beam can be selected after beam sweeping [33–37].

To be specific, define ${\mathcal {C}} = \lbrace {\bf c}_{\bar{n}}\rbrace _{{\bar{n}}=1,\ldots ,\bar{N}}$ as the codebook for $\bf v$, where $\bar{N}$ is the number of RIS beams and ${\bf c}_{\bar{n}}\in \mathbb {C}^{N \times 1}$ is the ${\bar{n}}$th RIS beam. Take the uplink as an example. Within the channel coherence time, the pilot model corresponding to the ${\bar{n}}$th RIS beam can be expressed as


(2) \begin{eqnarray*} {\bf Y}_{\bar{n}} = \sum _{k=1}^K \sqrt{P_k} ( {\bf d}_k+{\bf H}_2 {\rm diag}\lbrace {\bf c}_{\bar{n}}\rbrace {\bf h}_{1,k} ){\bf s}_k^H +{\bf Z}_{\bar{n}}, \end{eqnarray*}

where ${\bf Y}_{\bar{n}}\in \mathbb {C}^{M\times K}$ is the pilot matrix received by the BS antennas, *K* is number of users as well as the length of the pilot sequence, *P_k_* is the transmit power of user *k*, ${\bf s}_k\in \mathbb {C}^{K\times 1}$ is the pilot sequence from user *k*, satisfying ‖**s**_*k*_‖^2^ = 1 and ${\bf s}_k^H {\bf s}_j=0$ for *k* ≠ *j* and ${\bf Z}_{\bar{n}}\in \mathbb {C}^{M\times K}$ is the complex Gaussian noise matrix with independent and identically distributed entries. By multiplying Equation (2) with **s**_*k*_, we can extract the pilot component from user *k* as


(3) \begin{eqnarray*} \underbrace{{\bf Y}_{\bar{n}}{\bf s}_k}_{{\bf y}_{k,{\bar{n}}}} = \sqrt{P_k} ( {\bf d}_k+{\bf H}_2 {\rm diag}\lbrace {\bf c}_{\bar{n}}\rbrace {\bf h}_{1,k} ) + \underbrace{{\bf Z}_{\bar{n}}{\bf s}_k}_{{\bf z}_{k,{\bar{n}}}}. \end{eqnarray*}

The optimal RIS beam for user *k* with index ${\bar{n}}_k^*$ is the one that maximizes the pilot reception power:


(4) \begin{eqnarray*} {\bar{n}}_k^* = \arg \max _{{\bar{n}}=1,\ldots ,\bar{N}} \Vert {\bf y}_{k,{\bar{n}}}\Vert ^2. \end{eqnarray*}

Then, ${\bf c}_{{\bar{n}}_k^*}$ is selected as the RIS phase shift vector for user *k* in the data transmission phase and ${\bar{n}}_k^*$ is the implicit CSI we need to acquire.

Beam training is an efficient way to find a proper RIS phase shift design that can enhance the total channel quality. The computational complexity of beam training is $\mathcal {O}(KM\bar{N})$, much lower than that of explicit CSI estimation. Most importantly, we do not need to separate the direct link channel and the RIS link channel, and this method is not sensitive to the hardware impairments, such as imperfect phase shift and uncontrollable amplitude variations. However, the performance of beam training relies heavily on the codebook. On the one hand, a fine codebook that can seamlessly cover the whole service region of RIS is extremely powerful. On the other hand, the codebook that satisfies this requirement usually has a large size $\bar{N}$, resulting in a high beam training overhead of $K \bar{N}$ for *K* users.

The RIS codebook contains information of the spatial directions. If the user is equipped with multiple antennas as well, and only the line-of-sight (LoS) component exists in the RIS link, then the position of the user can be found with the beam training result [38,39].

### Blind beamforming

Apart from a beam index, when the RIS phase shift has limited resolution, a discrete phase shift index can be the implicit CSI we aim to acquire [40–42]. Denote the number of quantization bits of each RIS unit as *B*. A total of 2^*B*^ discrete phase shift values, denoted $\bar{\Theta }=\lbrace \bar{\theta }_1,\ldots , \bar{\theta }_{2^B} \rbrace$, can be constructed. When **h**_1, *k*_ and **H**_2_ are unknown to the RIS, a blind beamforming method is proposed to determine θ_1_, …, θ_*N*_ in Equation (1) from $\bar{\Theta }$ one by one. Similar to beam training above, a set of $\bar{N}$ RIS beams, also denoted by ${\mathcal {C}}$ here, is predefined. The difference is that random beams are utilized here, which means that during the beam sweeping phase, θ_1_, …, θ_*N*_ are independently and randomly generated from $\bar{\Theta }$. If $\bar{N}$ is large then, for the *n*th RIS unit, $\bar{\theta }_1, \ldots , \bar{\theta }_{2^B}$ are selected averagely. Take the selection of θ_*n*_ as an example. There are nearly ${\bar{N}}/{2^B}$ beams whose *n*th entry is $\bar{\theta }_1$, similar to $\bar{\theta }_2, \ldots , \bar{\theta }_{2^B}$. Collect all the RIS beams whose *n*th entry is $\bar{\theta }_b$ and calculate the average pilot reception power at the BS as


(5) \begin{eqnarray*} P_{n,b} = \mathbb {E}\lbrace \Vert {\bf y}_{k,\bar{n}}\Vert ^2 \vert \angle [{\bf c}_{\bar{n}}]_n = \bar{\theta }_b \rbrace . \end{eqnarray*}

Search for ${b_n^*}$ that achieves the highest pilot reception power at the BS:


(6) \begin{eqnarray*} b_n^* = \arg \max _{b=1,\ldots ,2^B} P_{n,b}. \end{eqnarray*}

Then, the discrete phase index $\bar{\theta }_{b_n^*}$ is chosen as the phase shift profile of the *n*th RIS unit. This is the conditional sample mean–based blind beamforming proposed in [41].

The blind beamforming method has an even lower requirement on the codebook, because a randomly generated codebook is acceptable. Similar to beam training, blind beamforming requires a training overhead of $K\bar{N}$, and is not sensitive to hardware impairments. Its computational complexity is $\mathcal {O}(KMN\bar{N} 2^B)$, and is thus higher than that of beam training. Blind beamforming has been experimentally verified to work well when *N* and *B* are small [41,42]. However, in order to guarantee that a proper ${b_n^*}$ can be selected for θ_*n*_, the number of generated random samples or the size of the codebook should still be large. With the user’s movement, blind beamforming may return outdated results, and the whole blind beamforming procedure should run again. Therefore, this method also suffers from the burden of high training overhead.

Implicit CSI acquisition is applicable to all categories of RISs. However, among them, IRS and active RIS-assisted mobile systems are more preferable to acquire implicit CSI since they are not required to have signal reception or processing capabilities. Furthermore, implicit CSI acquisition does not rely heavily on the RIS hardware characteristics. When the RIS has low cost and the hardware impairments cannot be ignored, it is preferable to directly search for a proper RIS phase shift design without estimating the explicit CSI.

## DOUBLE LINK CHANNEL SEPARATION

With the development of RIS manufacturing technology, the latest RISs have greatly improved hardware profile, resulting in the enhancement of explicit CSI acquisition robustness. Therefore, more attention has been paid to the estimation of explicit CSI, which refers to **h**_1, *k*_, **H**_2_ and their cascaded version. However, pilots in the direct link and in the RIS link are combined together at the receiver, making it difficult to discriminate them. In this section, we focus on the separation of channels in the two links. Commonly used RISs have been verified to offer reciprocity between uplink and downlink channels in TDD systems through experiments [43]. Considering that uplink training requires less pilot overhead than downlink training, we focus on the uplink when we study double link channel separation and the following channel estimation methods.

### ON/OFF RIS

An ON/OFF RIS is capable of reflecting and absorbing the incident wave, corresponding to the ON and OFF states, respectively [44,45]. When all RIS units are turned OFF, signals arriving at the RIS will be absorbed instead of being reflected, and, equivalently, we have **Φ** = **0**. The RIS link pilot component is completely diminished, solely resulting in the direct link pilot component in **y**_*k*_. Under this condition, **d**_*k*_ can be easily obtained.

Afterwards, by turning ON the RIS units one by one, we can sequentially estimate the channel on each RIS unit. Alternatively, by turning ON all RIS units but alternating the phase shift, the channel on all RIS units can be obtained simultaneously. Based on the fact that


(7) \begin{eqnarray*} {\bf H}_2 {\rm diag}({\bf v}){\bf h}_{1,k} = {\bf H}_2 {\rm diag}({\bf h}_{1,k}) {\bf v}, \end{eqnarray*}

the RIS phase shift vector **v** can be separated from the channel **H**_2_diag(**h**_1, *k*_). We define


(8) \begin{eqnarray*} {\bf G}_k \triangleq {\bf H}_2 {\rm diag}({\bf h}_{1,k}) \end{eqnarray*}

as the cascaded channel, satisfying ${\bf G}_k \in \mathbb {C}^{M \times N}$. Notably, the expression of **H**_2_diag(**h**_1, *k*_) requires that the user is equipped with only one antenna; otherwise, each user antenna corresponds to a distinct cascaded channel. When solely turning ON the *n*th RIS unit, we can estimate the cascaded channel on this unit, i.e. [**G**_*k*_]_:, *n*_. Owing to Equation (7), the RIS phase shift amount **v** has been separated from **H**_*k*_. Thus, with the estimate of **G**_*k*_, a proper **v** for data transmission can be designed.

The ON/OFF channel separation method requires *N* + 1 sets of RIS phase shift configurations. Note that a low-cost RIS usually has a large size, resulting in a large *N*. Furthermore, when *K* users exist, the total overhead, involving the length of pilot sequence **s**_*k*_ as well, should be *K*(*N* + 1). In other words, a long training period is required.

### ON-only RIS

When the absorption state is not supported by the RIS, i.e. the RIS only has the ON state, the RIS link pilot component always exists in **y**_*k*_. In order to separate the channels in the two links, special RIS phase shift designs should be applied [46–50]. In the training phase, assume that the *t*th RIS phase shift vector is ${\bf v}_t\in \mathbb {C}^{N \times 1}$. Then, by rewriting Equation (3), we get the uplink received pilot model from user *k* corresponding to **v**_*t*_ as


(9) \begin{eqnarray*} {\bf y}_{k,t} &= &\sqrt{P_k} ( {\bf d}_k+{\bf G}_k {\bf v}_t ) + {\bf z}_{k,t} \\ &=& \sqrt{P_k} [ {\bf d}_k\quad {\bf G}_k] {\left[\begin{array}{c}1 \\ {\bf v}_t \end{array}\right]} + {\bf z}_{k,t}. \end{eqnarray*}

Since the RIS phase shift vector **v**_*t*_ has been separated from **G**_*k*_ and can be known at the BS, we regard **v**_*t*_ as the pilot signal too. By stacking the pilot signals over *T* RIS phase shift vectors together into a matrix, we obtain


(10) \begin{eqnarray*} {\bf Y}_{k} = \sqrt{P_k} [ {\bf d}_k\quad {\bf G}_k] \left[{\begin{array}{c}{\bf 1}_{1\times T} \\ {\bf V} \end{array}}\right] + {\bf Z}_{k}, \end{eqnarray*}

where ${\bf V} = [{\bf v}_1,\ldots ,{\bf v}_T]\in \mathbb {C}^{N \times T}$.

Given Equation (10), we have two approaches to separate **d**_*k*_ and **G**_*k*_. One is to mathematically turn OFF the direct link. We now seek to find a matrix **V** that satisfies


(11) \begin{eqnarray*} \left[{\begin{array}{c}{\bf 1}_{1\times T} \\ {\bf V} \end{array}}\right] {\bf V}^\dagger = \left[{\begin{array}{c}{\bf 0}_{1\times N} \\ {\bf I}_N \end{array}}\right]. \end{eqnarray*}

Then, **G**_*k*_ can be obtained by calculating **Y**_*k*_**V**^†^. The other approach is to directly estimate the channels in the two links. Define


\begin{eqnarray*} \bar{{\bf G}}_k &=& [ {\bf d}_k,\quad {\bf G}_k]\in \mathbb {C}^{M \times (N+1)} \quad \text{and}\quad\\ \bar{\bf V}&=&\left[{\begin{array}{c}{\bf 1}_{1\times T} \\ {\bf V}\end{array}}\right] \in \mathbb {C}^{N \times T}. \end{eqnarray*}

Then, $\bar{{\bf G}}_k$ can be estimated through the widely used linear channel estimation methods, including least-square (LS) and linear minimum mean square error (LMMSE) schemes, if $\bar{{\bf V}}$ has full rank. A typical instance of $\bar{{\bf V}}$ that can be used in both approaches is an (*N* + 1)-dimensional discrete Fourier transformation (DFT) matrix, whose entries in the first row are all one. However, *T* = *N* + 1 is still required, indicating that the same amount of training overhead as using ON/OFF RIS is required. The computational complexity when $\bar{{\bf V}}$ is a DFT matrix is $\mathcal {O}(KMN)$.

The double link channel separation methods illustrated above are applicable to all RISs, especially IRSs and active RISs, but are not necessary to hybrid RISs. This is because a hybrid RIS has the ability to estimate the RIS-link channel and then separate the channels into two links with a greatly reduced pilot overhead, which is discussed in the subsequent section entitled ‘Individual channel estimation’.

## CASCADED CHANNEL ESTIMATION OVERHEAD REDUCTION

Because of the huge training overhead resulting from the linear estimation of the large-dimensional channel **G**_*k*_, we need to seek channel features for overhead reduction. Since **d**_*k*_ and **G**_*k*_ have already been separated, in this section, we only exploit the channel features in the RIS link that can help reduce the overhead for estimating the cascaded channel **G**_*k*_.

### Scaling law

According to the definition of **G**_*k*_ in Equation (8), for two distinct users *k* and *j*, we have


(12) \begin{eqnarray*} {\bf H}_2 {\rm diag}({\bf h}_{1,k}) = {\bf H}_2 {\rm diag}({\bf h}_{1,j}) \frac{{\rm diag}({\bf h}_{1,k})}{{\rm diag}({\bf h}_{1,j})}. \end{eqnarray*}

The scaling law between **G**_*k*_ and **G**_*j*_ can be found as follows [44,51,52]:


(13) \begin{eqnarray*} {\bf G}_k = {\bf G}_j {\rm diag} \bigg (\underbrace{ \frac{[{\bf h}_{1,k}]_1}{[{\bf h}_{1,j}]_1}, \ldots , \frac{[{\bf h}_{1,k}]_N}{[{\bf h}_{1,j}]_N} }_{\dot{\bf g}_{kj}\in \mathbb {C}^{1 \times N} }\bigg ). \end{eqnarray*}

That is, given **G**_*j*_, **G**_*k*_ can be obtained with the *N*-dimensional scaling channel $\dot{\bf g}_{kj}$. This is because **G**_*k*_ and **G**_*j*_ share the common BS-RIS channel **H**_2_. Recalling Equation (9) and omitting the direct link component, we have


(14) \begin{eqnarray*} {\bf y}_{k,t} &=& \sqrt{P_k}{\bf G}_k {\bf v}_t + {\bf z}_{k,t} \\ &=& \sqrt{P_k}{\bf G}_j {\rm diag}(\dot{\bf g}_{kj}){\bf v}_t + {\bf z}_{k,t} \\ &=& \sqrt{P_k}{\bf G}_j {\rm diag}({\bf v}_t)\dot{\bf g}_{kj} + {\bf z}_{k,t}. \end{eqnarray*}

Then, $\dot{\bf g}_{kj}$ can be linearly estimated given **y**_*k*_, **G**_*j*_ and the known **v**_*t*_. By exploiting the scaling law, the cascaded channel of the principle user **G**_1_ can be first estimated with the pilot overhead of *N*. Afterwards, the cascaded channels of other users **G**_*k*_ are obtained by simply estimating $\dot{\bf g}_{k1}, \, k=2,\ldots ,K$. Notably, in practical systems, we usually have *M* < *N*, causing a rank-deficient pseudo-inversion of **G**_*j*_. Under this condition, according to [44], a total of ⌈(*K* − 1)*N*/*M*⌉ instead of (*K* − 1)*N* pilots are needed to obtain $\dot{\bf g}_{k1},\, k=2,\ldots ,K$. Therefore, the total pilot overhead of multiuser double link channel estimation is greatly reduced from *K*(*N* + 1) to *N* + *K* + ⌈(*K* − 1)*N*/*M*⌉. The computational complexity is $\mathcal {O}(KMN^3)$.

### Channel sparsity

When the mobile system works in high frequencies, such as in the millimeter wave or even terahertz frequency bands, the channel experiences significant sparsity if the number of BS antennas or that of the RIS units grows large. Each individual channel is composed of a limited number of propagation paths, and each path can be described using a small amount of spatial parameters. Take the uniform linear array (ULA) as an example. Suppose that the RIS units are arranged in an ULA, and so are the BS antennas. When user *k* is in the far field of the RIS, **h**_1, *k*_ can be expressed as [53]


(15) \begin{eqnarray*} {\bf h}_{1,k} = \sum _{l=1}^{L_{1,k}} \alpha _{l,k} {\bf a}_N(\sin \phi _{l,k}), \end{eqnarray*}

where *L*_1, *k*_ is the number of paths in **h**_1, *k*_, satisfying *L*_1, *k*_ ≪ *N*, α_*l, k*_ is the complex path gain of the *l*th path, φ_*l, k*_ is the angle of arrival (AoA) of the *l*th path at RIS, while


(16) \begin{eqnarray*} {\bf a}_N(\sin \phi ) = [e^{j2\pi ({d}/{\lambda })\sin \phi },\ldots ,e^{j2\pi N({d}/{\lambda })\sin \phi }]^T \end{eqnarray*}

is the steering vector of an *N*-element ULA, *d* is the distance between two adjacent ULA elements and λ is the carrier wavelength. Similarly, in the far-field condition, **H**_2_ can be modeled as


(17) \begin{eqnarray*} {\bf H}_2 = \sum _{l=1}^{L_2} \beta _l {\bf a}_M(\sin \psi _l) {\bf a}_N^T(\sin \varphi _l), \end{eqnarray*}

where *L*_2_ is the number of paths in **H**_2_, satisfying *L*_2_ ≪ {*M, N*}, β_*l*_ is the complex path gain of the *l*th path, ψ_*l*_ is the AoA of the *l*th path at the BS and ϕ_*l*_ is the AoD of the *l*th path at the RIS. By applying Equations (15) and (17) in Equation (8), the cascaded channel has the structure


(18) \begin{eqnarray*} {\bf G}_k &=& \sum _{l_1=1}^{L_{1,k}} \sum _{l_2=1}^{L_2} \alpha _{l_1,k} \beta _{l_2} {\bf a}_M(\sin \psi _{l_2}) {\bf a}_N^T \\ &&\times \ (\sin \phi _{l_1,k} + \sin \varphi _{l_2}). \end{eqnarray*}

Define $\tilde{\bf G}_k = {\bf U}_M^H {\bf G}_k {\bf U}_N$, where **U**_*M*_ and **U**_*N*_ are *M*- and *N*-dimensional DFT matrices. Given that *L*_1, *k*_*L*_2_ ≪ *MN*, $\tilde{\bf G}_k$ is a sparse matrix with only a few entries having non-vanishing amplitude. Ignoring the direct link component, Equation (10) can be rewritten as


(19) \begin{eqnarray*} {\bf Y}_{k}^H{\bf U}_N^H = \sqrt{P_k}{\bf V}^H {\bf U}_M^H \tilde{\bf G}_k^H + {\bf Z}_k^H{\bf U}_N^H. \end{eqnarray*}

By regarding ${\bf V}^H {\bf U}_M^H$ as a measurement matrix, the sparse channel matrix $\tilde{\bf G}_k$ can be estimated through compressed sensing or deep learning, which requires greatly reduced training overhead [23,51,54,55]. Alternatively, given the structured channel model in Equation (18), **G**_*k*_ can be reconstructed using the spatial parameters, including $\alpha _{l_1,k} \beta _{l_2}$, $\sin \psi _{l_2}$ and $\sin \phi _{l_1,k}+\sin \theta _{l_2}$ [56–62]. Then, the channel reconstruction problem is translated to a parameter estimation problem, which also requires only a small amount of training overhead. Denote the average sparsity of $\tilde{\bf G}_k,\, k=1,\ldots , K$, as $\bar{L}$, satisfying $\bar{L} \ll N$. The total overhead approximates $K(1+\bar{L})$, and the computational complexity is generally $\mathcal {O}(KM^2\ N^2\bar{L})$.

**Figure 3.**
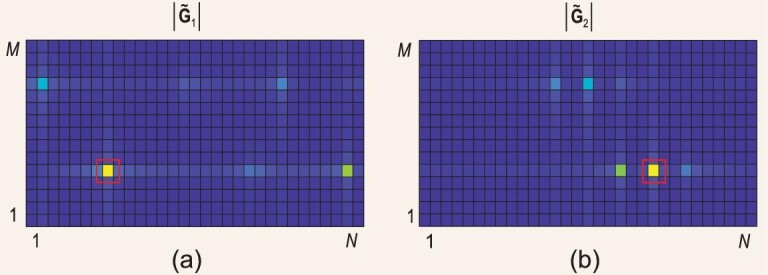
Common row sparsity between cascaded channels of (a) user 1 and (b) user 2. Red boxes frame the LoS paths in two channels.

Furthermore, because the *L*_1, *k*_*L*_2_ paths in **G**_*k*_ are stemming from *L*_1, *k*_ + *L*_2_ practical paths in **h**_1, *k*_ and **H**_2_, the multiuser cascaded channels $\tilde{\bf G}_k,\, k=1,\ldots , K$, hold common row sparsity[51]. Figure 3 illustrates the modulo of cascaded channel matrices of two users, i.e. $| \tilde{\bf G}_1|$ and $| \tilde{\bf G}_2|$. The biggest proportion of power of $| \tilde{\bf G}_1|$ is captured by the same rows that capture the major proportion of power of $| \tilde{\bf G}_2|$, demonstrating the common row sparsity between $\tilde{\bf G}_1$ and $\tilde{\bf G}_2$. The common sparsity and the channel scaling both result from the common channel **H**_2_. Exploring the common sparsity among multiple users can further reduce the pilot overhead and the computational complexity.

The cascaded channel is usually estimated in IRS and active RIS-assisted mobile systems. Nevertheless, for a hybrid RIS, since individual channels can be estimated, it is not necessary to estimate the cascaded channel.

## INDIVIDUAL CHANNEL ESTIMATION

Under some special channel conditions in an IRS or active RIS-assisted system, as well as in a hybrid RIS-assisted system, the individual channels **H**_2_ and **h**_1, *k*_ can be separated.

### IRS and active RIS

Since the IRS and the active RIS do not have signal processing capabilities, whilst the channel estimation can be performed only at the BS or user side, it is not easy to obtain the individual channels or separate them from the cascaded one. To be specific, recalling the scaling law in Equation (12), we may take **H**_2_diag(**h**_1, *j*_) and $\dot{\bf h}_{kj}$ to be **H**_2_ and **h**_1, *j*_, respectively, causing severe estimation errors. Moreover, from the multipath cascaded channel model in Equation (18), we see that the spatial angles that can be estimated by the BS are $\sin \psi _{l_2}$ and $\sin \phi _{l_1,k}+\sin \theta _{l_2}$. The AoA at the BS $\sin \psi _{l_2}$ can be clearly distinguished. The AoA and AoD at the RIS are added together, forming $\sin \phi _{l_1,k}+\sin \theta _{l_2}$. We cannot separate $\sin \phi _{l_1,k}$ and $\sin \theta _{l_2}$ from their superposition without any prior information. Therefore, it is not easy to separate **H**_2_ and **h**_1, *k*_ from **G**_*k*_.

However, if partial CSI is known in advance then it becomes possible to correctly separate **H**_2_ and **h**_1, *k*_. A LoS path usually exists between the BS and the RIS to guarantee a good performance of the latter. Recalling the channel model of **H**_2_ in Equation (17), let the first path be the LoS path. The LoS path has the strongest power, satisfying |β_1_| ≥ |β_*l*_| for *l* > 1. The expression of β_1_ can be further derived from the free-space propagation path loss model [63]. The angles of the LoS path, i.e. ψ_1_ and θ_1_, are determined by the locations of the BS and the RIS and known in advance when the BS and the RIS are fixed [58,64,65]. Recall the example in Fig. 3. If sin ψ_1_ and sin θ_1_ are fixed then $\sin \phi _{l_1,k}$ can be determined from $\sin \phi _{l_1,k}+\sin \theta _{1}$ for *l*_1_ = 1, …, *L*_1, *k*_. Moreover, given β_1_, it is easy to derive $\alpha _{l_1,k}$ from $\alpha _{l_1,k} \beta _{1}$. That is, all the spatial parameters in **h**_1, *k*_ have been successfully obtained, and **h**_1, *k*_ can be reconstructed by applying $\alpha _{l_1,k}, \sin \phi _{l_1,k}, \, l_1=1,\ldots ,L_{1,k}$, in Equation (15). Then, **H**_2_ can be extracted from **H**_2_diag(**h**_1, *k*_) given **h**_1, *k*_. Following the parameter estimation approach, in this condition, the total pilot overhead of estimating the double link channels approximates $K(1+\bar{L}_1)+L_2$, where $\bar{L}_1$ is the average sparsity of **h**_1, *k*_, *k* = 1, …, *K*, satisfying $\bar{L}_1 \ll N$. Hence, the computational complexity is about $\mathcal {O}(KM N^2 \bar{L}_1 + M^2 N^2 L_2)$.

Another way to separate the individual channels in the RIS link is to perform the parallel factor (PARAFAC) decomposition [66,67], which belongs to multiway analysis. By stacking **h**_1, *k*_, *k* = 1, …, *K*, together into a matrix, we have ${\bf H}_1 = [{\bf h}_{1,1}, \ldots , {\bf h}_{1,K}] \in \mathbb {C}^{N \times K}$. Given **v**_*t*_, the effective channel is defined as


(20) \begin{eqnarray*} {\bf H}_{{\rm eff},t} = {\bf H}_2 {\rm diag}({\bf v}_t){\bf H}_1 \in \mathbb {C}^{M \times K}, \end{eqnarray*}

where


(21) \begin{eqnarray*} [{\bf H}_{{\rm eff},t}]_{m,k} = \sum _{n=1}^N [{\bf H}_2]_{m,n} [{\bf H}_1]_{n,k} [{\bf V}]_{n,t}. \end{eqnarray*}

A three-way matrix ${\bf H}_{\rm eff} \in \mathbb {C}^{M \times K \times T}$ can be formulated, which has the following forms:


(22) \begin{eqnarray*} {\bf H}_{\rm eff}^1 = \left( {\bf H}_1^T \circ {\bf V}^T \right){\bf H}_2^T \in \mathbb {C}^{KT \times M}, \end{eqnarray*}

(23) \begin{eqnarray*} {\bf H}_{\rm eff}^2 = ( {\bf V}^T \circ {\bf H}_2 ){\bf H}_1 \in \mathbb {C}^{MT \times K}. \end{eqnarray*}

Given a random initial value, **H**_2_ and **H**_1_ can be iteratively and alternatively estimated from ${\bf H}_{\rm eff}^1$ and ${\bf H}_{\rm eff}^2$, respectively, through the linear estimation algorithms, such as LS. However, the PARAFAC decomposition-based individual channel separation method requires min(*M, K*) ≥ *N*, which is hard to achieve in practice. According to [66], the training overhead is in the range of [*K* + 2, *K* + *N*], and the computational complexity is $\mathcal {O}(KMN^2)$.

### Selection-type hybrid RIS

Hybrid RISs can make individual channel estimation easier to implement. For the selection-type hybrid RIS, we denote the channel between user *k* and the *N*_a_ active sensors and the channel between user *k* and the *N* − *N*_a_ passive units as ${\bf h}_{{\rm a},1,k}\in \mathbb {C}^{N_{\rm a}\times 1}$ and ${\bf h}_{{\rm p},1,k}\in \mathbb {C}^{(N-N_{\rm a})\times 1}$, respectively. When active sensors choose the antenna mode, the signal received by the active sensors from user *k* can be expressed as


(24) \begin{eqnarray*} {\bf y}_{{\rm a},1,k} = \sqrt{P_k}{\bf h}_{{\rm a},1,k}+ {\bf z}_{{\rm a},1,k}. \end{eqnarray*}

Given **y**_a, 1, *k*_, we can directly estimate **h**_a, 1, *k*_. However, if the active sensors are fixed and satisfy *N*_a_ ≪ *N* then **h**_p, 1, *k*_ can be obtained only through extrapolation from **h**_a, 1, *k*_, which requires that the **h**_p, 1, *k*_ are correlated with the **h**_a, 1, *k*_. When **h**_1, *k*_ satisfies the model in Equation (15), **h**_a, 1, *k*_ and **h**_p, 1, *k*_ share the same set of spatial parameters α_*l, k*_, sin φ_*l, k*_, *l* = 1, …, *L*_1, *k*_, and, thus, are highly correlated [23,25,68]. By applying these parameters in Equation (15), the full channel **h**_1, *k*_ is reconstructed. Alternatively, exploring the sparse structure or directly employing neural networks, **h**_1, *k*_ can be obtained from **h**_a, 1, *k*_ as well. The training overhead of acquiring **h**_1, *k*_ is *K*.

Similarly, **H**_2_ can be reconstructed by the BS sending downlink pilots to the RIS. If the active sensors are further equipped with signal transmission RF chains then **H**_2_ can also be reconstructed by the RIS sending uplink pilots to the BS. Notably, pilot sequences from different BS antennas or RIS active sensors should be orthogonal to each other, causing a training overhead of *M* or *N*_a_. The second way to obtain **H**_2_ is to conduct estimation at the BS. Having estimated the cascaded channel **G**_*k*_, **H**_2_ can be acquired given **h**_1, *k*_. However, the estimation of **G**_*k*_ needs a training overhead of $\sum _{k=1}^K \bar{L}_k$, because the RIS should alter the phase shift profile. Take the estimation of both-side individual channels at the RIS as an instance. The total pilot overhead of acquiring **H**_2_, **h**_1, *k*_ and **d**_*k*_ for *k* = 1, …, *K* is 2*K* + *M*, and the computational complexity is $\mathcal {O}(KM+KN_{\rm a}^2\bar{L}_1+M^2 N_{\rm a}^2 L_2)$.

### Beamforming-type hybrid RIS

For the beamforming-type hybrid RIS architecture, each RIS unit has the opportunity to be connected with a RF chain. Therefore, it is possible to make full channel estimation instead of channel extrapolation. However, since *N*_RF_ ≪ *N*, an analog beam sweeping stage is required, similar to hybrid beamforming systems [27,28]. We still take the estimation of **h**_1, *k*_ as an example. At time instance *t*, when the uplink pilot from user *k* arrives at the RIS, we have


(25) \begin{eqnarray*} {\bf y}_{{\rm b},1,k,t} = {\bf W}_t(\sqrt{P_k}{\bf h}_{1,k} + {\bf z}_{{\rm b},1,k,t}), \end{eqnarray*}

where ${\bf y}_{{\rm b},1,k,t}\in \mathbb {C}^{N_{\rm RF} \times 1}$ is the received pilot across the *N*_RF_ RF chains, ${\bf W}_t = [{\bf w}_{1,t},\ldots ,{\bf w}_{N_{\rm RF},t}]^T \in \mathbb {C}^{N_{\rm RF} \times N}$ contains *N*_RF_ RIS reception beams, ${\bf w}_{m,t}\in \mathbb {C}^{N \times 1}$ is the RIS reception beam in the *m*th RF chain in time instance *t*, $[{\bf w}_{m,t}]_n=\sqrt{\rho _{n,t}} e^{j\vartheta _{m,n,t}}$ if the *n*th RIS unit is connected with the *m*th RF chain, where ρ_*n, t*_ ∈ [0, 1] is the power ratio of the received signal over the incident signal and ϑ_*m, n, t*_ ∈ [0, 2π] is the reception phase shift, and [**w**_*m, t*_]_*n*_ = 0 otherwise. After collecting pilots in ⌈*N*/*N*_RF_⌉ time instances, the *N*-dimensional channel **h**_1, *k*_ can be linearly estimated. If **h**_1, *k*_ is sparse and satisfies the multipath model in Equation (15) then the training overhead can be further reduced through compressed sensing or parameter estimation methods.

Channel **H**_2_ can be obtained at the RIS by using downlink pilots sent from the BS in similar approaches, and the pilot sequences from different BS antennas should be orthogonal to each other. Alternatively, we can also utilize the uplink pilot components reflected by the RIS and received by the BS, which in time instance *t* is mathematically expressed as


(26) \begin{eqnarray*} {\bf y}_{{\rm b},2,k,t} = \sqrt{P_k} {\bf G}_k {\bf v}_t + {\bf z}_{{\rm b},2,k,t}, \end{eqnarray*}

where the *n*th entry of the reflection phase shift vector ${\bf v}_t \in \mathbb {C}^{N \times 1}$ satisfies $[{\bf v}_t]_n = \sqrt{1-\rho _{n,t}} e^{j\theta _{n,t}}$. Then, **H**_2_ can be estimated following the same approaches as that for the selection-type hybrid RISs. Similarly, take the linear estimation of both-side individual channels at RIS as an example, and suppose that **W**_*t*_ is in the DFT format. The total pilot overhead of acquiring **H**_2_, **h**_1, *k*_ and **d**_*k*_ for *k* = 1, …, *K* is *K*[1 + (1 + *M*)⌈*N*/*N*_RF_⌉], and the computational complexity is $\mathcal {O}(KMN)$.

### Double time scales

In a typical RIS-assisted mobile communication system, the locations of the BS and the RIS are fixed. The BS is generally mounted at the top of a building, and the RIS is then deployed in the LoS region of the BS to guarantee a strong link between them. The environment between the BS and the RIS is relatively stable, and thus the coherence time of **H**_2_, denoted as *T*_2_, is long. On the other hand, the mobile user has a high probability to keep moving. The coherence time of **h**_1, *k*_, denoted *T*_1, *k*_, is much shorter than *T*_2_. Therefore, channel estimation can be performed in double time scales [69–71]. For longer *T*_2_, the estimation of **H**_2_ only needs to be performed once. Within this period, the estimation of **h**_1, *k*_ is performed in every shorter period of *T*_1, *k*_. Since the training overhead required by the estimation of **h**_1, *k*_ is limited when **H**_2_ is given, the overall overhead of double time scale training can be very low.

## CHANNEL ESTIMATION ALGORITHMS

A comprehensive comparison of the overhead and complexity of the above mentioned existing CSI acquisition methods is provided in Table 1. Moreover, Table 2 summarizes the applicability of these methods to different categories of RISs.

**Table 1.** Overhead and complexity of existing CSI acquisition methods for acquiring **H**_2_, **h**_1, *k*_, and **d**_*k*_, *k* = 1, …, *K*.

CSI acquisition method		Overhead	Complexity
Implicit CSI acquisition	Beam training	$K \bar{N}$	$\mathcal {O}(KM\bar{N})$
	Blind beamforming	$K \bar{N}$	$\mathcal {O}(KMN\bar{N} 2^B)$
Double link channel separation	ON/OFF RIS	*K*(*N* + 1)	$\mathcal {O}(KMN)$
	ON-only RIS	*K*(*N* + 1)	$\mathcal {O}(KMN)$
Cascaded channel estimation	Scaling law	$K+N+\big \lceil \frac{(K-1)N}{M}\big \rceil$	$\mathcal {O}(KMN^3)$
	Channel sparsity	$K(1+\bar{L})$	$\mathcal {O}(KM^2\ N^2\bar{L})$
Individual channel estimation	IRS/active RIS (LoS)	$K(1+\bar{L})+L_2$	$\mathcal {O}(KM N^2\bar{L}_1 + M^2\ N^2 L_2)$
	Selection type	2*K* + *M*	$\mathcal {O}(KM+KN_{\rm a}^2\bar{L}_1+M^2 N_{\rm a}^2 L_2)$
	Beamforming type	$K\big [1+(1+M)\big \lceil \frac{N}{N_{\rm RF}}\big \rceil \big ]$	$\mathcal {O}(KMN)$

**Table 2.** Applicability of existing CSI acquisition methods to different categories of RISs.

		Hyrbid RIS	
CSI acquisition method	IRS/active RIS	Selection type	Beamforming type
Implicit CSI acquisition	Preferable	Not necessary	Not necessary
Double link channel separation	Preferable	Not necessary	Not necessary
Cascaded channel estimation	Preferable	Not necessary	Not necessary
Individual channel estimation	Conditional	Preferable	Preferable

In previous sections, we briefly mentioned the algorithms that are used to estimate the channels or their parameters, including linear estimation, compressed sensing, parameter estimation and deep learning. This section will provide more details about them in the context of RIS-assisted mobile communication systems.

### Linear estimation

Linear estimation is the most classic and widely used estimation method in practical systems [72]. The computational complexity is fixed and the estimation accuracy is stable. Linear estimation methods do not rely on channel features or structures like Equation (15) or (17). Referring back to Equation (10), which has the expression


(27) \begin{eqnarray*} {\bf Y}_{k} = \sqrt{P_k} \bar{\bf G}_k \bar{\bf V} + {\bf Z}_{k}, \end{eqnarray*}

the LS estimate of $\bar{\bf G}_k$ is


(28) \begin{eqnarray*} {{\hat{\bar{\bf G}}}_{{\rm LS},k}} = \frac{1}{\sqrt{P_k}} {{\bf Y}_{k}} \bar{\bf V}^\dagger, \end{eqnarray*}

and the LMMSE estimate is


(29) \begin{eqnarray*} {{\hat{\bar{\bf G}}}_{{\rm LMMSE},k}} = {\bf R}_{\bar{\bf G}_k}\! \bigg ( {\bf R}_{\bar{\bf G}_k} + \frac{\sigma _n^2}{P_k} {\bf I} \bigg )^{-1} {{\hat{\bar{\bf G}}}_{{\rm LS},k}}, \end{eqnarray*}

where ${\bf R}_{\bar{\bf G}_k}$ is the covariance matrix of the channel $\bar{\bf G}_k$ and $\sigma _n^2$ is the variance of the noise. Notably, $\bar{\bf V}$ should have full column rank, which requires a sufficient amount of pilot overhead [28]. Considering that LMMSE has much higher estimation accuracy than LS, it is more widely adopted for channel estimation in RIS-assisted systems [44,73,74].

### Compressed sensing

When the channels are sparse, compressed sensing can be applied for low-overhead channel estimation. Recall the signal model for compressed sensing-based cascaded channel estimation in Equation (19) such that $\tilde{\bf G}_k$ is the sparse cascaded channel in the angular domain to be estimated. We say that $\tilde{\bf G}_k$ is in the angular domain because $\tilde{\bf G}_k$ is built on the dictionaries **U**_*M*_ and **U**_*N*_, which simultaneously and uniformly sample and draw grids on the angles. Each entry of $\tilde{\bf G}_k$ represents the channel component on the corresponding sampled angular pair on the two-dimensional (2D) grid. Given Equation (19), the general problem to be solved by compressed sensing can be written as


(30) \begin{eqnarray*} &&\min\! \Vert \tilde{\bf G}_k \Vert _0 \\ \text{such that}&& \left\Vert {\bf Y}_{k}^H{\bf U}_N^H - \sqrt{P_k}{\bf V}^H {\bf U}_M^H \tilde{\bf G}_k^H \right\Vert _F^2 \le \epsilon ,\\ \end{eqnarray*}

where ε is a predefined residual threshold. The problem requires using the smallest amount of entries in $\tilde{\bf G}_k$ to capture the majority of information in the channel. Sparse channel representations and compressed sensing problems for hybrid RISs in Equations (24) and (25) can be expressed in a similar way.

Since the sparsity, i.e. the number of distinct entries in $\tilde{\bf G}_k$, is unknown in advance, and in order to avoid the huge computational complexity caused by exhaustive search, a typical solution is the greedy algorithm such as orthogonal match pursuit (OMP) [23,75,76]. By inspecting the problem in Equation (30), other algorithms such as the alternating direction method of multipliers [25,27,77] can also recover the sparse channel.

In a multiuser scenario, the scaling law as well as the common sparsity between **G**_*k*_ and **G**_*j*_ can be exploited in compressed sensing-based channel estimation [51,75,76]. By writing **G**_*j*_ as


(31) \begin{eqnarray*} {\bf G}_j = {\bf U}_M^H \tilde{\bf G}_k {\bf U}_N {\rm diag}\ (\dot{\bf g}_{jk}), \end{eqnarray*}

we see that users share a common sparse base $\tilde{\bf G}_1$, which can be recovered through the multiple measurement vector-based compressed sensing estimator for accuracy enhancement.

### Super-resolution parameter estimation

Unlike compressed sensing, which describes the angles using a grid defined by the dictionary, super-resolution parameter estimation aims to obtain more precise angular estimates that are no longer limited by the grids. We still take the cascaded channel as an example. Let **G**_*k*_ = **G**_*k*_(**Θ**_*k*_), where ${\boldsymbol \Theta }_k = [\sin \psi _1, \ldots , \sin \psi _{L_2}, \sin \phi _{1,k} + \sin \theta _{1}, \ldots , \sin \phi _{L_1,k} + \sin \theta _{L_2}]$ contains the angles to be estimated. We now rewrite Equation (10), by ignoring the direct link, as


(32) \begin{eqnarray*} {\bf Y}_{k}^H = \sqrt{P_k}{\bf V}^H{\bf G}_k({\boldsymbol \Theta }_k)^H + {\bf Z}_k^H. \end{eqnarray*}

We seek to estimate **Θ**_*k*_ from ${\bf Y}_{k}^H$. For example, maximum likelihood (ML) estimation finds the angles that satisfy [56,57]


(33) \begin{eqnarray*} \hat{\boldsymbol {\Theta} }_k = \stackrel{\displaystyle\rm arg\, min}{\boldsymbol \Theta } \left\Vert {\bf Y}_{k}^H - \sqrt{P_k}{\bf V}^H{\bf G}_k({\boldsymbol \Theta })^H \right\Vert _F^2. \\ \end{eqnarray*}

Apart from ML, classic and widely used super-resolution parameter estimation algorithms include multiple signal classification, estimation of signal parameters via rotational invariance techniques, etc. [78–80]. Even though these algorithms have different objective functions, a super-resolution grid is generally required. This grid can be much denser than the grid in compressed sensing methods. By searching over the grid, one grid point that can satisfy the objective function is chosen as the estimate.

If the grid density is not high then a further step is conducted to refine the on-grid estimate towards the one that is closer to the real angle value. Under this condition, enhancement of the parameter estimation accuracy can be achieved on basis of the on-grid estimates obtained by compressed sensing. For example, a gradient descent step can be added after the grid-based matching in each iteration of OMP [59,61]. Alternatively, a Newton refinement step can also refine the on-grid angle estimates towards their real values [58]. Newton refinement integrated with OMP forms the Newtonized orthogonal matching pursuit algorithm [81], which has been widely applied in sparse channel reconstruction [82–84]. Another compressed sensing-based approach, which can overcome the on-grid effect, is atomic norm minimization [39,60,68,85], where the grid is composed of infinite cardinality.

Acquisition of the spatial parameters not only supports low-overhead sparse channel reconstruction, but enables further applications such as user or target localization [38,39,56,57].

### Deep learning

Deep learning has experienced rapid development in recent years. When applied to channel estimation, deep learning does not heavily rely on a precise channel model like Equation (15), (17) or (18). In RIS-assisted systems, deep learning can be used to estimate the cascaded channel or the individual channels for both the IRS and hybrid RIS.

In an IRS-assisted system, deep learning is usually adopted to estimate the cascaded channel **G**_*k*_. By directly regarding the received pilots at the BS or user side as the input of the neural network, the estimate of **G**_*k*_ can serve as the output of a convolutional neural network [86,87]. Alternatively, deep learning can be introduced to further enhance the accuracy of LS or LMMSE cascaded channel estimation results. By regarding it as a denoising problem, a deep residual network can be applied to fix this problem [88,89]. The denoising concept was also applied in [90]. Different from Liu *et al.* [88,89], Jin *et al.* [90] regarded the channel matrix as an image. Three practical residual neural networks were adopted to improve the cascaded channel estimation accuracy, while the network input was the received pilot matrix.

Deep learning-based channel estimation is also preferable in hybrid RIS-assisted systems. For the selection-type hybrid RIS especially, since pilots can be received by a small amount of active units, only the sampled channel on these units can be directly estimated. Then, the full channel can be extrapolated through a residual network. The input of the network is the sampled channel, and the output is the full channel [24]. The denoising approach can be further applied here. Considering that the accuracy of the full channel reconstruction from a few observations on the active sensors is limited, a denoising neural network can be employed to refine the channel reconstruction result [55].

## OPEN PROBLEMS

From the review above, we see that extensive research has been carried out on CSI acquisition in traditional RIS-assisted mobile communication scenarios. In future mobile systems, the emergence of new architectures and new application scenarios of RISs entails new challenges. In this section, we discuss two open problems that have to be addressed in the context of CSI acquisition in future RIS-assisted systems.

### Near-field effect

A RIS has the attractive advantages of low cost and low power consumption. Thus, we can increase the size of a RIS to further improve wireless communication services. However, as the RIS grows large, the user or the BS falls into the near field of the large RIS. Then, the near-field effect kicks in [64,91–94]. Under this condition, in the multipath channel model, each path is described by the 2D or 3D position of the user, BS or scatterer, instead of their direction or angle as in the far-field channel. The near-field steering vector ${\bf a}_N(r,\theta )\in \mathbb {C}^{N\times 1}$ is different from the far-field one, whose *n*th entry can be expressed as


(34) \begin{eqnarray*} [{\bf a}_N(r,\theta )]_n = \frac{\lambda }{4\pi D_n(r_l,\theta _l)} e^{j2\pi {D_n(r_l,\theta _l)}/{\lambda }}, \end{eqnarray*}

where (*r*, θ) is the polar position of the user, BS or scatterer, and *D_n_*(*r_l_*, θ_*l*_) is the distance between the user, BS or scatterer and the *n*th RIS unit. Take **h**_1, *k*_ as an example. The near-field channel **h**_1, *k*_ can be modeled as


(35) \begin{eqnarray*} {\bf h}_{1,k} = \sum _{l=1}^{L_{1,k}} \beta _l {\bf a}_N(r_l,\theta _l). \end{eqnarray*}

Note that the sparsity of a near-field channel is not always apparent under the DFT transformation. When the user is quite close to the RIS, a large angular spread can be observed even if only a single LoS path exists in **h**_1, *k*_. Compressed sensing and super-resolution parameter estimation should be adjusted to cater for the near-field effect. In particular, the channel parameters to be estimated become (*r_l_*, θ_*l*_), *l* = 1, …, *L*_1, *k*_. Moreover, by exploring these parameters, user localization can be achieved if the LoS path exists.

### High-mobility scenarios

High-mobility wireless communications, including high-speed trains and unmanned aerial vehicles (UAVs) scenarios, have gained increasing attention over the past years. For the purpose of enhancing the coverage and providing seamless wireless services with low cost, the introduction of RISs have been considered in these high-mobility systems [95–98]. However, since trains and UAVs move fast, the environment and the channel vary rapidly. Wireless communication in high-mobility scenarios suffers from a severe Doppler effect and the channel aging problem [98]. In IRS-assisted systems especially, channel estimation in the RIS link becomes even more challenging. Hence, how to efficiently obtain CSI with limited pilot overhead is of great importance.

Fortunately, the property of double time scales can also be exploited in high-mobility scenarios. Take the high-speed train scenario as an example. The RIS can be deployed on one side of the railway, coated on the window of the train, or on the wall or roof of the train [95,99]. If the location of the RIS is fixed then **H**_2_ varies slowly and has a much longer channel coherence time than **h**_1, *k*_ and **d**_*k*_. On the other hand, if the RIS moves with a high-speed train then the coherence time of **h**_1, *k*_ becomes longer than that of **H**_2_ and **d**_*k*_, which is different from the double time scales in the formal case. Equally important, temporal correlation exists among channels in continuous time slots [98]. CSI obtained in previous time slots can be utilized to track the fast-varying channel in time with reduced pilot overhead. Furthermore, the Doppler effect can be mitigated by leveraging the time–frequency space framework [100]. However, the fast handover in high-mobility scenarios is an issue that cannot be ignored, introducing challenges to CSI acquisition in RIS-assisted systems.

## CONCLUSIONS

This article provided a comprehensive review of the state of the art on CSI acquisition in RIS-assisted mobile communication systems. Newly appeared categories of RISs, including selection-type and beamforming-type hybrid RISs as well as active RISs, were considered. By classifying CSI into implicit CSI and explicit CSI, we surveyed the acquisition of these two types of CSI. Acquisition of explicit CSI was studied via a step-by-step approach, from double link channel separation, low-overhead cascaded channel estimation, individual channel estimation to the estimation algorithms. The review ended with two open problems in future RIS-assisted systems. We articulated that, with the emergence of more advanced RISs and algorithms, CSI acquisition in RIS-assisted mobile systems will experience new breakthroughs.
